# Harnessing Radiomics and Explainable AI for the Classification of Usual and Nonspecific Interstitial Pneumonia

**DOI:** 10.3390/jcm14144934

**Published:** 2025-07-11

**Authors:** Turkey Refaee, Ouf Aloofy, Khalid Alduraibi, Wael Ageeli, Ali Alyami, Rafat Mohtasib, Naif Majrashi, Philippe Lambin

**Affiliations:** 1 Department of Diagnostic Radiography Technology, Faculty of Nursing and Health Sciences, Jazan University, Jazan 85145, Saudi Arabia; wageeli@jazanu.edu.sa (W.A.); aalmansour@jazanu.edu.sa (A.A.); nmajrashi@jazanu.edu.sa (N.M.); 2 Department of Radiology, King Faisal Specialist Hospital & Research Center, Riyadh 11211, Saudi Arabia; oufaloofy@gmail.com (O.A.); khalid.alduraibi@gmail.com (K.A.); 3 Biomedical Physics Department, King Faisal Specialist Hospital & Research Center, Riyadh 11211, Saudi Arabia; rafatmohtasib@gmail.com; 4 Department of Medical School, Alfaisal University, Riyadh 11533, Saudi Arabia; 5 The D-Lab, Department of Precision Medicine, GROW-School for Oncology and Reproduction, Maastricht University, 6200 MD Maastricht, The Netherlands; philippe.lambin@maastrichtuniversity.nl; 6 Department of Radiology and Nuclear Medicine, Maastricht University Medical Center, 6200 MD Maastricht, The Netherlands

**Keywords:** usual interstitial pneumonia, nonspecific interstitial pneumonia, high-resolution computed tomography, radiomics, SHAP analysis

## Abstract

**Objectives**: Accurate differentiation between usual interstitial pneumonia (UIP) and nonspecific interstitial pneumonia (NSIP) is crucial for guiding treatment in interstitial lung diseases (ILDs). This study evaluates the efficacy of clinical, radiomic, and combined models in classifying UIP and NSIP using high-resolution computed tomography (HRCT) scans. **Materials and Methods**: A retrospective analysis was performed on 105 HRCT scans (UIP = 60, NSIP = 45) from Faisal Hospital and Research Center. Demographic and pulmonary function data formed the clinical model. Radiomic features, extracted using the pyRadiomics package, were refined using recursive feature elimination. A combined model was developed by integrating clinical and radiomic features to assess their complementary diagnostic value. Model performance was assessed via the area under the receiver operating characteristic curve (AUC). SHapley Additive exPlanations (SHAP) analysis, including both global feature importance and individual-level explanations, was used to interpret the model predictions. **Results**: The clinical model achieved an AUC of 0.62 with a sensitivity of 54% and a specificity of 78%. The radiomic model outperformed it with an AUC of 0.90 with a sensitivity and specificity above 85%. The combined model showed an AUC of 0.86 with a sensitivity of 88% and a specificity of 78%. SHAP analysis identified texture-based features, such as GLCM_Idmn and NGTDM_Contrast, as influential for classification. **Conclusions:** Radiomic features enhance classification accuracy for UIP and NSIP compared to clinical models. Integrating HCR into clinical workflows may reduce variability and improve diagnostic accuracy in ILD. Future studies should validate findings using larger, multicenter datasets.

## 1. Introduction

Interstitial lung diseases (ILDs) represent a heterogeneous group of pulmonary disorders characterized by varying degrees of inflammation and fibrosis within the lung interstitium [1,2]. Among these, usual interstitial pneumonia (UIP) and nonspecific interstitial pneumonia (NSIP) are two critical subtypes that have garnered significant clinical attention [3]. UIP is most commonly associated with idiopathic pulmonary fibrosis (IPF), a progressive and often fatal condition, while NSIP is frequently linked to autoimmune diseases and has a generally better prognosis [4]. The differentiation between these two patterns is not merely academic; it carries profound implications for patient management, treatment strategies, and overall outcomes in affected individuals [5,6].

The accurate classification of UIP versus NSIP is essential for effective patient management and prognostication. Studies have shown that misclassification can lead to inappropriate treatment decisions, which may adversely affect patient survival and quality of life. For example, while UIP typically necessitates antifibrotic therapies due to its aggressive nature, NSIP may benefit from immunosuppressive treatments [7,8]. The ability to distinguish between these two patterns can significantly influence clinical decisions, underscoring the importance of precise diagnostic methodologies in ILD management [9]. In clinical practice, this diagnostic precision is often achieved through multidisciplinary discussion (MDD), which integrates clinical, radiologic, and histopathologic data [10]. MDD is particularly valuable in cases with discordant imaging and biopsy findings; however, its implementation may vary across institutions depending on the availability of expert teams. This variability can lead to delays or inconsistencies in diagnosis, highlighting the need for scalable, reproducible tools to support decision-making.

Given the central role of imaging in MDD, high-resolution computed tomography (HRCT) has become the cornerstone in the diagnostic evaluation of ILDs, providing detailed imaging that reveals the specific patterns associated with UIP and NSIP [10]. The role of HRCT in diagnosing these conditions cannot be overstated; it not only aids in identifying the disease but also helps in monitoring disease progression over time.

Despite the advantages of HRCT, radiologists face significant challenges when differentiating between UIP and NSIP. Interobserver variability remains a notable issue, with studies indicating that even experienced radiologists may arrive at differing conclusions based on the same imaging data [4,11,12]. This variability can lead to inconsistent diagnoses, further complicating patient management. As such, there is a pressing need for more objective methods to enhance diagnostic accuracy in this context.

Artificial intelligence is gaining popularity, driven by the growing volume of imaging data and the availability of computational resources [13]. Various types of AI exist, including traditional machine learning and deep learning approaches. However, challenges such as data availability, generalizability across imaging protocols, and clinical integration still limit widespread adoption. Alongside these AI developments, quantitative imaging techniques have rapidly gained traction in medical research, offering new avenues for data-driven analysis [14]. Handcrafted radiomics (HCR) represents an innovative approach that holds promise for improving the classification accuracy of UIP and NSIP. In HCR, quantitative imaging features are manually engineered using predefined mathematical formulas applied to HRCT scans, rather than learned automatically from data [14,15]. This technique allows for the analysis of texture, shape, and intensity variations within lung tissue, potentially identifying subtle differences that are not readily apparent to the human eye. Recent studies have demonstrated that HCR can effectively differentiate between various ILD subtypes, suggesting that this methodology could significantly reduce interobserver variability. When combined with machine learning and explainable AI techniques, HCR may further enhance classification accuracy and support clinical decision-making [16,17,18].

This study evaluates the effectiveness of HCR in differentiating UIP from NSIP patterns in HRCT scans, aiming to assess its potential for improving diagnostic accuracy and enhancing patient care in these pulmonary disorders.

## 2. Materials and Methods

### 2.1. Patient

**Figure 1:**
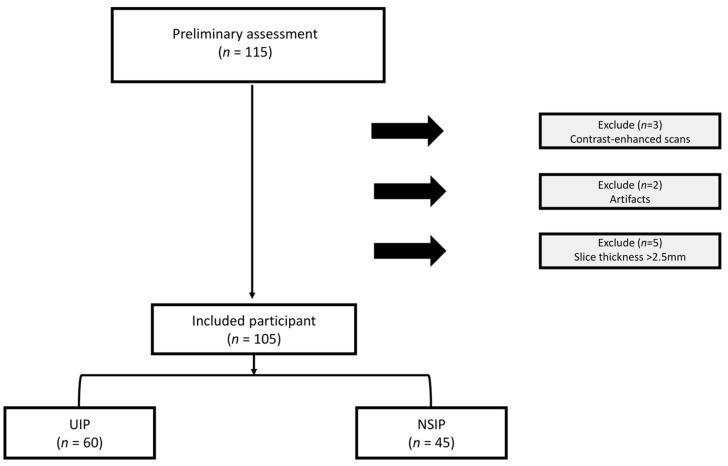
The flowchart diagram shows the patient selection process. UIP, usual interstitial pneumonia, NSIP, nonspecific interstitial pneumonia.

The study was approved by the Institutional Review Board (IRB) committee of King Faisal Hospital and Research Center (KFHRC) (approval number: 2221265; approval date: 22 January 2023). Informed consent was waived due to the retrospective nature of the study. The study retrospectively analyzed a total of 115 HRCT scans (UIP = 60, NSIP = 55) from KFHRC, with one scan per patient, collected between 2019 and 2022. To be included, scans had to be non-contrast enhanced and have slice thicknesses ≤ 2.5 mm. Exclusion criteria encompassed contrast-enhanced images, scans with metal or motion artifacts, and those with slice thicknesses exceeding 2.5 mm. Figure 1 shows the patient selection process. All diagnoses were confirmed through MDD. For each participant, researchers collected demographic information, clinical data, and pulmonary function test results. This included age, sex, body mass index (BMI), forced expiratory volume in 1 s (FEV1), and forced vital capacity (FVC).

### 2.2. Image Acquisition and Segmentation

The high-resolution computed tomography (HRCT) scans were performed at the same medical facility. CT scans were performed using GE Medical Systems and Siemens CT scanners across the dataset. Sharp kernels such as LUNG and Bl57d were employed, along with iterative reconstruction techniques to optimize image quality. Scanning parameters included a tube voltage of 120 kV and varying mAs settings, with values ranging from 249 to 747 mAs, adjusted based on patient-specific needs. The slice thickness ranged from 0.5 mm to 2.5 mm, allowing for high-resolution imaging where necessary to capture fine anatomical details while balancing scan time and radiation exposure across all cases. For the segmentation of the entire lung, an automated workflow was employed, developed using MIM software (MIM Software Inc., Cleveland, OH, USA). The accuracy and consistency of this automated segmentation approach have been validated in prior studies [19]. In our study, all segmentations were visually reviewed by an experienced thoracic radiologist as part of a quality control protocol. Any segmentation errors or inaccuracies identified during this review were manually corrected using the editing tools provided within the MIM software.

### 2.3. Data Imputation and Data Split

To handle missing data in our dataset, particularly for the FEV1 and FVC variables, we utilized the Multiple Imputation by Chained Equations (MICE) method. MICE is an advanced approach that addresses missing data by applying a series of regression models where each variable with missing values is predicted based on the others in the dataset. We selected this technique for its versatility in managing different types of variables and its capability to reflect the uncertainty of imputed values by generating multiple complete datasets [20]. Our dataset included 60 patients with UIP and 45 patients with NSIP. Missing values were notable, with 19 UIP patients (31.7%) and 4 NSIP patients (8.9%) having incomplete data for FVC and FEV1. Due to the limited size of our dataset and the need to retain all available data, we opted for imputation since removing patients with missing values was not feasible. Specifically, we created three imputations, resulting in three complete datasets, under the assumption that the data were Missing At Random (MAR). This iterative imputation process maintained the original data distribution [21]. The quality of the imputed data was assessed using a Student’s *t*-test to compare the distributions of the original and imputed values, ensuring the imputation did not introduce significant bias or alter the data’s underlying distribution. Additionally, we used density plots to visualize the distribution of the original and imputed data, providing a graphical assessment of the imputation’s impact.

After performing MICE to complete our dataset, we proceeded with data splitting. To ensure patient-level independence and prevent data leakage, we used the GroupShuffleSplit method from scikit-learn, specifying each patient’s unique identifier as the grouping variable [22]. The random seed was set to 42 to guarantee reproducibility. The dataset was divided into a training set comprising 80% of the data and a testing set with the remaining 20%. This 80:20 split is widely adopted in machine learning research, providing a balance between robust model training and sufficient data for unbiased model evaluation, especially in moderate-sized datasets such as ours (*n* = 105) [23]. Although GroupShuffleSplit (version 1.3) does not enforce strict stratification by class label, we verified that the proportions of UIP and NSIP patients were similar in both the training and testing sets after the split. This approach ensured that each subset retained a representative distribution of patients within each group. Additionally, a Wilcoxon rank-sum test (*p* ≤ 0.05) was applied to confirm there were no significant differences between the subsets across clinical features.

### 2.4. HCR Feature Extraction

**Figure 2:**
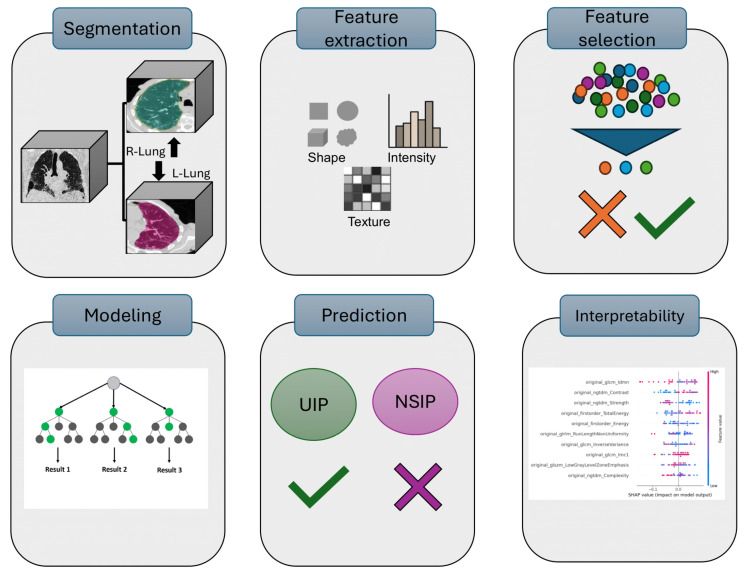
HCR workflow for classifying UIP and NSIP from HRCT scans. The process begins with lung segmentation from CT images, followed by HCR feature extraction using pyRadiomics. Next, feature selection methods are applied to select the most informative set of features. The selected features are then used to train a Random Forest classifier to arrive at a prediction. Finally, SHAP analysis is performed to interpret the model’s predictions and feature contributions.

To ensure consistency and comparability across images, all CT scans were resampled to an isotropic voxel size of 1 mm × 1 mm × 1 mm using third-order B-spline interpolation, as implemented in pyRadiomics (version 3.0.1). This interpolator was chosen for its ability to preserve spatial relationships while minimizing interpolation artifacts, in alignment with IBSI recommendations for radiomic studies. For feature extraction, we employed pyRadiomics, an open-source Python package compliant with the Image Biomarker Standardization Initiative (IBSI) [24,25]. While the IBSI recommends standardized preprocessing workflows, we intentionally preserved native Hounsfield Unit (HU) values without additional intensity normalization (e.g., z-score or histogram matching). This decision was guided by the IBSI’s acknowledgment that CT HU values represent standardized physical measurements when acquired under consistent protocols. To reduce noise and mitigate inter-scanner variability, we discretized the voxel intensities into 25 bins of Hounsfield Units. This binning process helps to stabilize feature extraction and improves the comparability of features across different CT scanners and acquisition parameters. The extracted features describe first-order statistics (*n* = 18), shape-based features (*n* = 14), the Gray-Level Co-Occurrence Matrix (GLCM, *n* = 22), the Gray-Level Run Length Matrix (GLRLM, *n* = 16), the Gray-Level Size Zone Matrix (GLSZM, *n* = 16), the Gray-Level Dependence Matrix (GLDM, *n* = 14), and the Neighboring Gray Tone Difference Matrix (NGTDM, *n* = 5). A workflow for HCR from segmentation to data analysis is illustrated in Figure 2.

### 2.5. Feature Selection and Model Development

To maintain the integrity of our model evaluation and minimize the risk of overfitting, all feature selection and model training procedures were conducted exclusively on the training dataset. Our feature selection process followed a multi-step approach aimed at reducing dimensionality and identifying the most relevant radiomic features for distinguishing between UIP and NSIP patterns. Initially, we addressed multicollinearity by identifying and removing highly correlated features. Specifically, feature pairs with a Spearman correlation coefficient (r) of 0.90 or higher were considered highly correlated, and the feature with the highest average correlation to all others was eliminated [26]. The remaining features were further refined using recursive feature elimination (RFE) with a Random Forest classifier on the subset selected after applying Spearman’s correlation coefficient. RFE was performed using 5-fold cross-validation to assess the classification accuracy, and the 10 features that yielded the highest accuracy were selected for the final model. Ultimately, these top 10 features were retained for inclusion in the final Random Forest model. The Random Forest classifier was implemented using 100 decision trees and a fixed random seed, while all other parameters were left at default values. The Random Forest algorithm was chosen for its strong performance in HCR-based classification tasks and its robustness in handling high-dimensional imaging data [27,28].

Three distinct models were developed: a clinical model, an HCR model, and a combined model incorporating both clinical and radiomic features. This modeling framework was designed to evaluate the diagnostic value of each data source independently and in combination, allowing us to assess the unique and additive contributions of clinical and radiomic features. This approach not only facilitates a transparent comparison of model performance but also helps identify whether radiomics provides complementary information beyond what is captured by standard clinical variables. The clinical model was constructed using five routinely available variables: age, sex, BMI, FVC, and FEV1. These features were used without additional transformation or dimensionality reduction. The HCR model was built using the top 10 features selected through recursive feature elimination (RFE) following correlation-based filtering. To construct the combined (composite) model, we concatenated the selected clinical and radiomic features into a unified feature set and applied the same Random Forest training pipeline. All three models shared the same architecture, hyperparameters, and evaluation strategy to allow for a fair and direct performance comparison. To further assess the potential for overfitting and the likelihood of identifying spurious correlations within the classification pipeline, we performed a randomization test by shuffling outcome labels and repeating the entire modeling process 1000 times. This approach allowed us to evaluate the model’s performance under conditions where no real association should exist.

### 2.6. Model Explanation and Visualization

SHapley Additive exPlanations (SHAP) analysis is a post hoc interpretability technique based on game theory that quantifies how individual features contribute to a model’s prediction [29,30]. In this study, we used the TreeExplainer from the SHAP Python package (v0.44.0) to interpret the output of the trained Random Forest models. SHAP values were computed using the held-out test dataset, focusing on the positive class (UIP) to analyze model decisions relevant to disease classification.

We generated summary plots to visualize global feature importance and waterfall plots to explain individual predictions by illustrating the additive contribution of each feature. This approach provided both dataset-level and case-level interpretability, enhancing transparency and clinical relevance.

### 2.7. Radiomics Quality Score, TRIPOD Guidelines, and Statistical Analysis

To ensure the quality and reproducibility of our HCR study, we utilized the Radiomics Quality Score (RQS) framework [31]. This comprehensive assessment tool, designed specifically for radiomics studies, consists of 16 items with varying scores that sum to a maximum of 36 points. This study adhered to the Transparent Reporting of a Multivariable Prediction Model for Individual Prognosis or Diagnosis (TRIPOD) guidelines [32]. All statistical analyses were conducted using Python (version 3.12). For comparing datasets, we employed non-parametric methods to account for potential non-normal distributions in our data. Continuous variables were analyzed using the Wilcoxon rank-sum test, which assesses whether two independent samples come from the same distribution. For categorical variables, we utilized X2 Fisher’s exact test for small sample sizes, evaluating the association between categorical variables. The performance of our classification model was primarily assessed using the area under the curve (AUC) of the receiver operating characteristic (ROC) analysis. We report 95% confidence intervals (CI) for the AUC to quantify the precision of our estimate. To evaluate the models’ goodness of fit, we employed the Hosmer–Lemeshow test, which assesses whether the observed event rates match expected event rates in subgroups of the model population [33]. Additionally, we generated calibration plots to visually inspect the agreement between predicted probabilities and observed outcomes, providing insight into the model’s reliability across different probability ranges. We also conducted a decision curve analysis to evaluate the net benefits of our models across various threshold probabilities.

## 3. Results

### 3.1. Patient Characteristics

After applying the exclusion criteria, a total of 105 patients were enrolled in the study, including 60 diagnosed with UIP and 45 diagnosed with NSIP. The demographic and clinical characteristics of the NSIP and UIP groups are summarized in Table 1. Significant differences were observed in age (*p* < 0.001), sex distribution (*p* < 0.001), and BMI (*p* = 0.035), with UIP patients being older and predominantly male, and having a lower BMI compared to those with NSIP. No significant differences were found in FEV1 (*p* = 0.227), while FVC showed a borderline significance (*p* = 0.052).

Wilcoxon rank-sum tests revealed no significant differences between the training and testing datasets for most variables, including age (*p* = 0.314), sex (*p* = 0.116), FVC (*p* = 0.678), and FEV1 (*p* = 0.184). However, a statistically significant difference was observed in BMI (*p* = 0.046), indicating a slight variation in BMI values between the two sets at the *p* ≤ 0.05 level. In evaluating the imputed data, *t*-test results showed no significant differences between the original and imputed data for UIP, with *p*-values of 0.9 for FVC and 0.7 for FEV1, and similar results for NSIP, where both FVC and FEV1 also had *p*-values of 0.9. These non-significant *p*-values confirm that the imputation process did not introduce any significant bias or alter the underlying distribution of the data. Density plots illustrating the distribution of both the original and imputed data can be found in Supplementary Figure S1.

### 3.2. Feature Extraction and Feature Selection

We initially extracted 105 original features from the complete dataset, excluding 14 shape-related features. Our feature selection process involved two main steps: first, removing highly correlated features, and second, applying the RFE algorithm. The clinical model used 4 features, the HCR model incorporated 10 features, and the combined model utilized 14 features. These model-specific feature sets were then used as inputs for the respective group comparisons. The comprehensive list of selected features for each model is provided in the Supplementary Material (Table S1).

### 3.3. Performance of the Models

**Figure 3:**
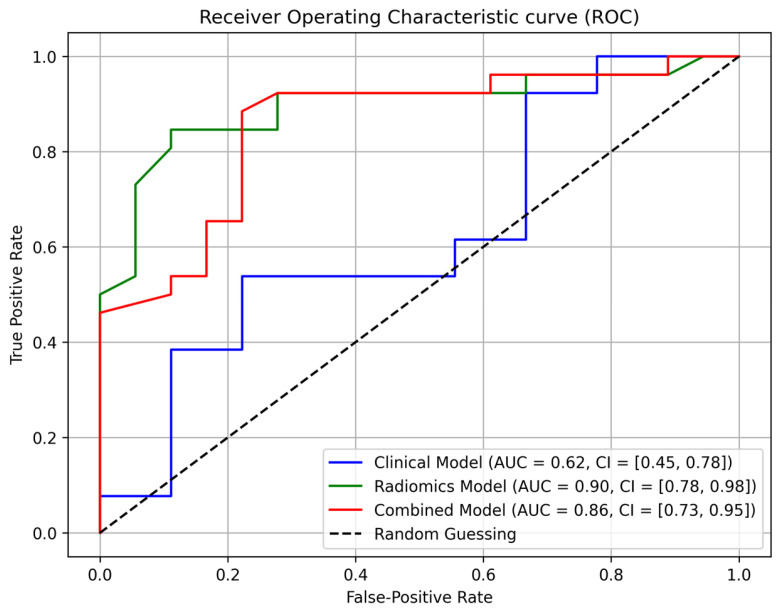
Area under the receiver operating characteristic (AUC) curve of different models for the test set.

In this study, we developed and evaluated three distinct machine learning models: a clinical model, an HCR model, and a combined model. All models achieved an AUC of 1.00 on the training dataset, indicating perfect classification within the training set. On the test dataset, the clinical model demonstrated an AUC of 0.62 (95% CI: 0.45–0.78), while the HCR model exhibited enhanced performance, attaining an AUC of 0.90 (95% CI: 0.78–0.98). The combined model, which integrates both clinical and radiomic features, achieved an AUC of 0.86 (95% CI: 0.73–0.95) (Figure 3). To optimize model performance, decision thresholds were determined using Youden’s index on the training data, resulting in thresholds of 0.75, 0.48, and 0.45 for the clinical, HCR, and combined models, respectively. These performance metrics are summarized in Table 2.

After 1000 iterations with randomized outcome labels, the mean AUC was 0.5 for both HCR and combined models, indicating that the models performed no better than random chance. This result confirms that the pipeline does not inherently identify spurious correlations and that the model’s predictive power in the original analysis was not due to overfitting.

### 3.4. Explanation and Visualization of Radiomic Models

**Figure 4:**
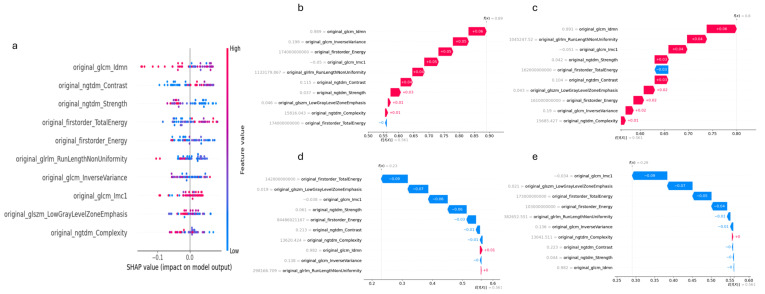
(**a**) SHAP global summary plot, (**b**–**e**) SHAP waterfall plots for four test cases that have UIP or NSIP. Red color indicates features that increase the prediction (positive SHAP value), blue color indicates features that decrease the prediction (negative SHAP value).

The SHAP summary plot (Figure 4a) highlights the most significant features that influenced the model’s predictions for differentiating UIP from NSIP. GLCM_Idmn consistently demonstrated the highest impact, contributing positively towards UIP predictions. NGTDM_Contrast and NGTDM_Strength also played substantial roles, with both features influencing the model to varying degrees depending on the instance. First-order_TotalEnergy and GLRLM_RunLengthNon-Uniformity showed more variable effects but were still key in pushing predictions toward either UIP or NSIP. Overall, texture-based features such as GLCM Idmn and NGTDM Contrast were the most reliable in classifying UIP, while other metrics like GLCM Inverse Variance and first-order Energy provided additional refinement in the model’s output.

To further explore the model’s predictive behavior, SHAP waterfall plots were generated for two UIP cases and two NSIP cases (Figure 4b–e). In UIP Instance 1 (Figure 4b), the model predicted UIP with a high probability of 0.89, driven by positive contributions from GLCM Idmn (+0.06), first-order Energy (+0.05), and NGTDM Contrast (+0.04), while a minor negative effect from GLCM Imc1 (−0.05) had minimal impact on the final prediction. For UIP Instance 2 (Figure 4c), the model also showed high confidence with a prediction probability of 0.80, primarily influenced by GLCM Idmn (+0.06) and GLRLM Run Length Non-Uniformity (+0.04), with a small negative contribution from GLCM Imc1 (−0.03) being outweighed by the positive influences.

In NSIP Instance 1 (Figure 4d), the model assigned a low UIP probability of 0.23, strongly influenced by negative contributions from first-order Total Energy (−0.09) and GLSZM Low Gray-Level Zone Emphasis (−0.07), pushing the prediction toward NSIP. Minor positive contributions, such as that of GLCM Idmn, were insufficient to offset the negative impact. Similarly, in NSIP Instance 2 (Figure 4e), with a UIP probability of 0.29, first-order Total Energy (−0.07) and GLCM Imc1 (−0.09) had strong negative effects, reinforcing the NSIP classification, while small positive contributions from NGTDM Contrast and GLCM Idmn were insufficient to alter the outcome.

### 3.5. RQS, TRIPOD, Decision Curve Analysis, and Calibration

**Figure 5:**
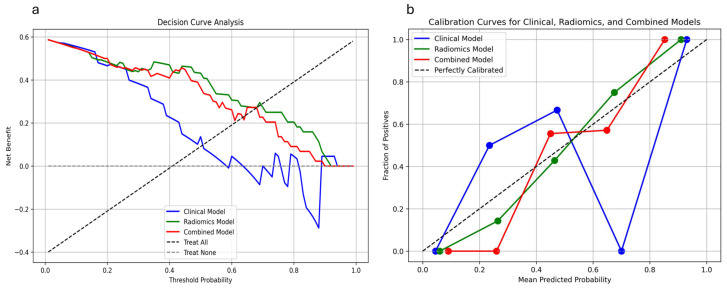
(**a**) Decision curve analysis of the clinical model, the HCR model, and the combined model for the test set. (**b**) Calibration curves of the clinical model, the HCR model, and the combined model for the test set.

The total RQS achieved for this study was 14 out of a possible 36 points, resulting in a score of 38.89% (Supplementary Table S2). The reporting of the model development, evaluation metrics, and participant characteristics follows the TRIPOD-AI guidelines. A completed TRIPOD-AI checklist is provided in the Supplementary Materials Table S3. The decision curve analysis (DCA) shows that the HCR model provided the highest net benefit across a wide range of threshold probabilities compared to the other models. Specifically, it performed well between the threshold probabilities of 0.1 to 0.7, where its net benefit was consistently higher than that of the clinical model and close to or surpassing that of the combined model (Figure 5a). The HCR and combined models exhibited strong calibration, as evidenced by the *p*-values obtained from the Hosmer–Lemeshow test. Specifically, the HCR model yielded a *p*-value of 0.81, and the combined model recorded a *p*-value of 0.73. The HCR model consistently showed the highest net benefit across most threshold probabilities, especially between 0.2 and 0.8, indicating strong clinical value for decision support (Figure 5b).

## 4. Discussion

In this study, we proposed an HCR-based approach to differentiate UIP and NSIP which demonstrated significant improvements in diagnostic accuracy compared to clinical models. We also performed SHAP analysis to explain the predictions of radiomic models. Our HCR model achieved a high AUC, outperforming the clinical model and closely matching the combined model. Our findings suggest that HCR holds significant potential as a valuable tool to support clinical decision-making, potentially reducing the time required by radiologists and enhancing diagnostic accuracy.

The clinical model, which relied solely on traditional clinical parameters, demonstrated a relatively modest performance, with an AUC of 0.62, a sensitivity of 0.54, and a specificity of 0.78. This performance underscores the inherent challenges in differentiating between UIP and NSIP using clinical data alone. The low sensitivity suggests that the clinical model may miss a significant number of UIP cases, despite its relatively high specificity, which indicates its ability to reliably exclude non-UIP cases. In contrast, the HCR model, which leveraged advanced imaging features extracted from CT scans, exhibited a superior performance, achieving an AUC of 0.90, significantly higher than the clinical model. This improvement was accompanied by a balanced sensitivity of 0.85 and specificity of 0.89, further highlighting the HCR model’s ability to accurately differentiate between UIP and NSIP. These results align with the growing body of evidence supporting the utility of HCR in capturing subtle textural and morphological differences in lung tissue, which are often undetectable through clinical examination alone. The enhanced performance of the HCR model emphasizes its potential to overcome the limitations of traditional clinical approaches in ILD diagnosis. Notably, the combined model showed slightly lower performance than the HCR-only model, likely because the clinical features provided limited additional predictive value beyond what was already captured by the HCR. This suggests that radiomic features may inherently encode structural correlates of physiological changes, resulting in partial redundancy when combined with clinical variables.

Our findings are strongly supported by several recent studies that have explored the use of HCR in differentiating between UIP and NSIP, as well as other interstitial lung diseases (ILDs). For instance, Chen et al. (2023) achieved an AUC of 0.952 for the training set and 0.838 for the test set when distinguishing UIP from NSIP using lung texture features extracted from CT images [17]. Similarly, Refaee et al. (2022) demonstrated that an HCR model could achieve an AUC of 0.85 for the classification of IPF and non-IPF ILDs, supporting the robustness of texture-based radiomic features in these contexts [6,16]. These results are comparable to the AUC of 0.90 achieved in our study, underscoring the effectiveness of HCR in capturing subtle textural and morphological differences that are often missed by conventional clinical evaluation. The consistency across these studies highlights the potential of HCR as a valuable tool for improving diagnostic accuracy in ILDs, further validating the clinical relevance of our findings.

The integration of SHAP analysis provided a detailed understanding of the HCR model’s performance in differentiating UIP from NSIP. The SHAP summary plot revealed that GLCM Idmn and NGTDM Contrast were the most impactful features, consistently contributing to the accurate classification of UIP. These texture-based metrics likely capture the complex structural characteristics associated with fibrotic patterns in UIP, aligning well with the known pathophysiological differences between UIP and NSIP. In particular, GLCM Idmn measures local texture homogeneity. Its positive contribution to UIP predictions may reflect the structured, repetitive appearance of the dense fibrosis and honeycombing seen in advanced UIP, which can create locally uniform textures in HRCT. In contrast, NSIP typically presents with diffuse inflammation and ground-glass opacity, resulting in more fine-grained variability and lower GLCM Idmn values [34]. Positive SHAP contributions from GLCM Idmn and first-order Energy in UIP cases highlighted their critical role in pushing predictions toward UIP, indicating the model’s reliance on these specific texture variations for accurate classification.

Conversely, negative SHAP contributions from first-order Total Energy and GLSZM Low Gray-Level Zone Emphasis were significant in NSIP cases, suggesting that these features are more representative of NSIP’s distinct imaging profile. The case-specific SHAP waterfall plots underscore the robustness of the model’s interpretability by illustrating how individual feature contributions influenced predictions. This local interpretability is essential for clinical applications, as it provides clarity on why the model arrived at certain classifications, ultimately enhancing trust in AI-assisted diagnostics. By combining global insights with case-specific explanations, the SHAP analysis demonstrated that the model’s decision-making process aligns with clinical expectations, confirming its potential as a supplementary tool for radiologists. The ability to understand the importance of features and their direction of influence is crucial for facilitating clinical adoption, as it provides transparency in model predictions. This study supports the value of incorporating advanced radiomic analysis, backed by explainable AI techniques, to improve diagnostic accuracy and consistency in distinguishing between UIP and NSIP. In addition, HCR-based predictions can be generated rapidly once imaging is available, offering a time-efficient and scalable alternative to the traditional diagnostic workflow. Unlike MDD, which requires coordinated input from multiple specialists and can lead to delays, HCR models provide consistent and immediate decision support, which is particularly valuable in settings with limited access to expert teams.

Despite the promising results, our study has several noteworthy limitations: First, the relatively small sample size (*n* = 105) may limit the generalizability of our findings, even though they align with the existing literature. This limitation stems partly from the rarity of NSIP, which restricted the number of available cases [35]. Given the exploratory nature of this work and the lack of standardized effect size estimates for radiomic features in ILD, a formal power analysis was not conducted. Second, the need for larger multicenter studies to confirm the robustness of the HCR model across diverse patient populations and imaging protocols highlights a limitation in the current study’s scope and generalizability. We are actively pursuing multicenter collaborations to address this in future research. Third, as a retrospective study, there is a risk of selection bias since we included only cases that met strict criteria, potentially limiting the model’s applicability in broader clinical settings. Additionally, the lack of external validation is a significant limitation; while our model performed well on our test set, validation using independent datasets from different institutions is crucial to confirm its real-world utility. These limitations underscore the need for further research to solidify the potential of HCR in distinguishing UIP from NSIP patterns in HRCT scans.

Future studies should prioritize external validation and assess model performance on HRCT scans from various manufacturers and protocols to ensure widespread applicability. Additionally, integrating deep learning approaches, such as convolutional neural networks (CNNs), with HCR may allow for automated learning of complex imaging features, potentially enhancing classification accuracy [36]. This approach could complement handcrafted radiomic features by capturing deeper, more nuanced patterns within high-resolution imaging data. Moreover, exploring advanced fusion strategies for combining clinical and radiomic data may further improve model performance in distinguishing UIP from NSIP.

## 5. Conclusions

In conclusion, this study demonstrates that an HCR-based approach—supported by a Random Forest classifier and enhanced by SHAP interpretability—can effectively differentiate between UIP and NSIP, offering a more objective and transparent diagnostic method. This approach has the potential to significantly improve clinical decision-making, reduce interobserver variability, and ultimately lead to better patient outcomes. Further research involving larger datasets and more advanced methodologies will be essential to fully harness the potential of HCR in the clinical management of ILDs.

## Figures and Tables

**Table 1:** Demographic and clinical information of the study participants. BMI: body mass index, FEV: forced expiratory volume, FVC, forced vital capacity.

Characteristic	NSIP (*n* = 45)	UIP (*n* = 60)	*p*-Value
Age, mean (SD)	51.24 (16.29)	64.01 (15.48)	<0.001
Sex, *n* (%)			
Female	34 (75.6)	22 (36.1)	<0.001
Male	11 (24.4)	38 (63.9)	
BMI, mean (SD)	30.84 (8.70)	28.82 (5.04)	0.035
FEV1, mean (SD)	61.29 (17.37)	58.51 (15.64)	0.227
FVC, mean (SD)	53.52 (14.76)	49.68 (13.47)	0.052

**Table 2:** Detailed predictive and diagnostic values of various models studied using the testing dataset.

Model	Accuracy %	Sensitivity %	Specificity %	PPV %	NPV %
Clinical	64	54	78	78	54
HCR	86	85	89	92	80
Combined	84	88	78	85	82

## Data Availability

The data utilized in this study are privately owned and are not publicly available due to confidentiality agreements and ownership restrictions. However, further details regarding the data may be provided upon reasonable request to the corresponding author, subject to approval by the data owners.

## Supplementary Materials

The following supporting information can be downloaded at https://www.mdpi.com/article/10.3390/jcm14144934/s1: Table S1: Name of the features in each model.; Table S2: Radiomics Quality Score (RQS).; Table S3: TRIPOD AI-Checklist; Figure S1: Density plots comparing the original and imputed data for FVC and FEV across UIP and NSIP subgroups. Panels A and B represent the density plots for FVC in UIP and NSIP, respectively, while panels C and D show the density plots for FEV in UIP and NSIP, respectively.
